# Editorial: Changes in the auditory brain following deafness, cochlear implantation, and auditory training, volume II

**DOI:** 10.3389/fnhum.2023.1124304

**Published:** 2023-02-06

**Authors:** Fawen Zhang, Ji-Hye Han, Ravi Samy, Jing Xiang

**Affiliations:** ^1^University of Cincinnati, Cincinnati, OH, United States; ^2^Hallym University, Chuncheon-si, Republic of Korea; ^3^Cincinnati Children's Hospital Medical Center, Cincinnati, OH, United States

**Keywords:** cochlear implantation, auditory training, deafness, electroencephalography, auditory evoked potentials

A cochlear implant (CI) is a prosthetic device used to treat severe to profound hearing loss. A CI bypasses the damaged inner ear and directly stimulates the auditory nerve with an array of electrodes, providing sound information. To achieve ideal speech perception outcomes, it is important to minimize the mismatch between the assigned frequency range to each electrode on the array and the frequency range naturally represented by the neural element stimulated by the electrode (frequency-to-place mismatch, Di Maro et al., 2022).

Neurologically, CI users' auditory system may display great variation in central auditory processing of sound features, which has been considered an important contributor to variability in CI outcomes (Liang et al., 2018; Han and Dimitrijevic, 2020). Examining cortical encoding of sound in CI users will result in better ways (e.g., the use of objective neural measures) to assess CI benefits. In this Research Topic, McGuire et al. used electroencephalography (EEG) to understand the cortical processing of frequency changes in CI users and its contribution to variability of CI outcomes measured behaviorally. The authors examined the scalp-recorded cortical acoustic change complex (ACC) evoked by the frequency change contained in a pure tone in adult CI users. Results showed that the latency values of ACC peak components were negatively correlated with behavioral performance on speech perception and frequency change detection. The variation in CI speech outcomes can be partially explained (about 16–20%) by the variability of cortical encoding of frequency changes reflected by the ACC.

Perceptually, CI users typically show improved sound perception over time following CI activation. One debatable issue is whether the pitch percept also changes over time in CI ears (Reiss et al., 2007, 2015; Vermeire et al., 2015). CI users with normal hearing (NH) in the non-implanted ear are perfect participants to examine the possible pitch change in the CI ear, as they can use the NH ear to precisely judge the pitch of sound to the CI ear by interaural comparison. Dorman et al. recruited 5 such CI users who had been implanted with a short electrode array, which caused the frequencies represented by the neural element stimulated by the electrodes to be 1–2 octaves higher than the frequencies assigned to the electrodes. The participants were presented with a signal to the CI ear and then to the NH ear. They were asked how the signal to the NH ear should be changed to match the signal in the CI ear. Then the signal was manipulated until the participant said that the pitch was very close to that of the CI ear. Results showed 4/5 listeners needed an increase in frequency to match the sound of their CI ear at 8 months after CI activation and this upshift in pitch did not change at 35 months. Results did not support previously found phenomenon that pitch uplift would be less prominent over time as a result of cortical plasticity, possibly because the frequency-to-place mismatch was too large.

While CI users' brain can learn new modality of hearing after implantation *via* passive exposure to sound environment, active auditory training has been suggested to maximize CI benefits (Fu et al., 2008; Roman et al., 2016; Reis et al., 2021). Zhao et al. provided valuable direction of possibly using music training in CI users, who typically complain difficulty in music perception. The researchers used the EEG technique to record the frequency-following response (FFR), a neural representation of the pitch of complex sounds in the upper auditory brainstem, to a Mandarin lexical tone in 43 monolingual English-learning infants at 7 and 11 months of age. Infants were semi-randomly assigned to receive either no intervention or a 4-week music intervention involving active music listening and synchronizing body movements to the musical beats at 9 months of age between the two sessions of FFR testing. Results showed that the FFR pitch-tracking accuracy was resistant to decline that was seen in the group receiving no intervention, suggesting that music intervention during critical period can maintain the auditory systems' ability to track acoustic properties of non-native speech.

Effects of active training may be enhanced if selective attention, which is critical for speech understanding in noise (Lawrence et al., 2018; Price and Bidelman, 2021), is integrated, and if the subject receives a neurofeedback during the training. Kim et al. used a neurofeedback training paradigm to enhance the attentional modulation of cortical auditory evoked responses in NH listeners. This study provided a novel direction of possibly using the neurofeedback training approach in CI users. The authors randomly assigned young NH adults to either the Experimental or the Placebo Group to listen attentively to repeated trials of target words in a distractor noise. While the Placebo Group received a feedback based on participants' performance accuracy (“correct” and “incorrect”) in the behavioral task of selecting an utterance with a higher pitch, the Experimental Group received a visual feedback based on EEG responses (neurofeedback) that reflected the strength of attentional modulation on cortical auditory evoked responses during the training. After four sessions of training, the Experimental Group exhibited consistent improvement in the attentional modulation of the evoked responses to the training stimuli and a better performance in the SiN task, whereas the Placebo Group did not show such an effect.

In summary, the current collection of recently published papers discussed cortical encoding of frequency changes and if the pitch percept changes over time in CI users using a short electrode array. It also collected papers examining the effects of music training and neurofeedback-based selective attention training in NH listeners, which provide novel training approaches for CI users. We look forward to future editorial work that promotes the understanding of neurological changes in auditory system following deafness, cochlear implantation, auditory training, and neurofeedback training.

## Author contributions

FZ drafted this editorial and other authors provided feedback. All authors contributed to the article and approved the submitted version.
